# Courier Review Call Book

**Published:** 1887-01

**Authors:** 


COURIER-REVIEW CALL BOOK, a Physicians' Pocket Reference Book and Visiting List for '87: arranged and prepared by E. M. Nelson, M. D. Ph. D., Editor St. Louis Courier of Medicine; Physician to St. Louis Protestant Hospital; late Physician to St. Louis Maternity Hospital; etc. J. H. Chambers & Co., Publishers and Dealers in Medical Books, St. Louis, Mo.; Chicago, Ills, and Atlanta, Ga.

This is a most sensible pocket companion, it being large enough to contain the necessary entries without cramping, yet not too large to carry in pocket; it contains, in addition to the usual contents of such books, a diagnostic table of eruptive fevers, the treatment for poisoning, how to care for galvanic batteries, &c., &c.; of flexible morocco, and on good paper. It is a very desirable book to possess. The nicest thing of the kind we have yet seen.

